# Supplementary material for: LRRC46 Accumulates at the Midpiece of Sperm Flagella and Is Essential for Spermiogenesis and Male Fertility in Mouse
Source: Int J Mol Sci. 2022 Jul 31;23(15):8525. doi: 10.3390/ijms23158525 (PMC9369233; doi:10.3390/ijms23158525)
Supplement: Supplementary file 1 [file ijms-23-08525-s001.zip › ijms-1812647-supplementary.pdf]

Supplementary Materials

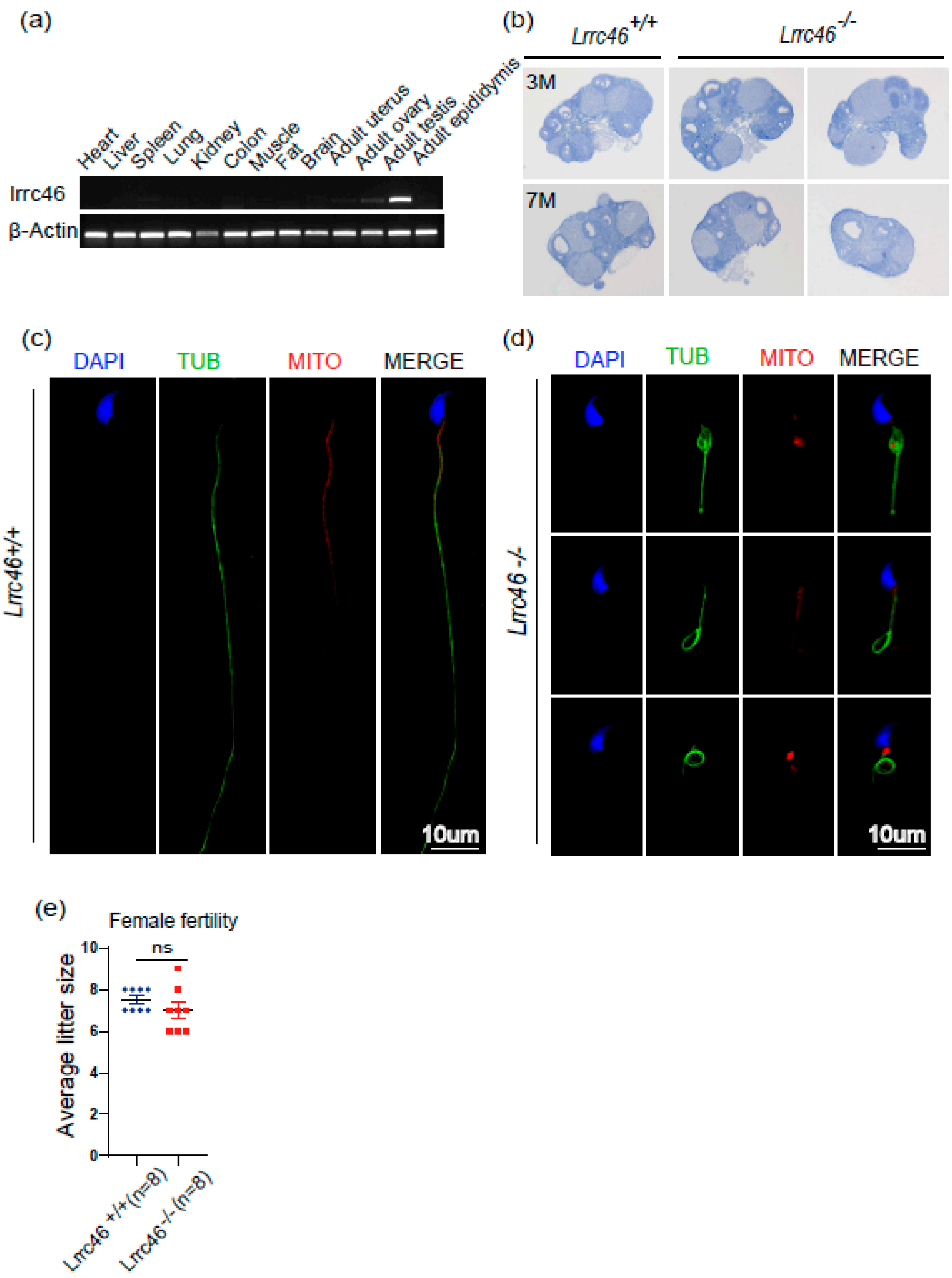

Figure S1: Absence of *Lrrc46* present no defect in female fertility, and defect the mitochondrial sheath. (**a**) Using RT-PCR, *Lrrc46* was predominately expressed in adult testis, weakly in adult ovary, but no expression in other

organs, including heart, liver, spleen, kidney, colon, muscle, fat and brain. (**b**) Hematoxylin-eosin staining of ovary sections from the *Lrrc46*$^{+/+}$ and *Lrrc46*$^{-/-}$ female mice. (**c**,**d**) The immunofluorescence analysis for α/β-tubulin (green) and Mito Tracker (red) was performed in *Lrrc46*$^{+/+}$ and *Lrrc46*$^{-/-}$male mice spermatozoa. The nucleus was stained with DAPI (blue). (**e**) The average litter size of *Lrrc46*$^{+/+}$ and *Lrrc46*$^{-/-}$ female mice in 3 months (n=8 independent experiments). KO females were completely fertile. Data are presented as the mean ± SD.

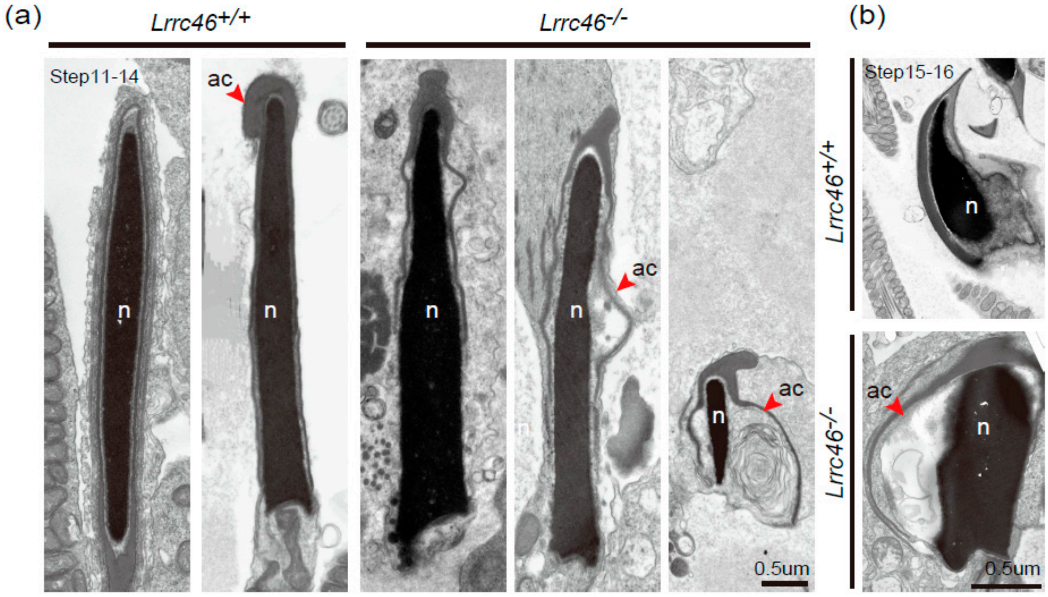

Figure S2: Absence of *Lrrc46* present sperm head acrosome defects. (**a**,**b**) Via transmission electron microscopy, spermatozoa from *Lrrc46*$^{-/-}$ male mice show head acrosome defects. TEM analysis revealed the detachment of the acrosome (ac) from the sperm nuclei (n) in the sperms from the *Lrrc46*$^{-/-}$ mice. The red arrowhead indicates the acrosome. The arrow indicates the plasma membrane (pm). Scale bar: 0.5um.
